# Annotations

**Published:** 1901-10-26

**Authors:** 


### ANNOTATIONS.

### The Storage of Thames Water.

The question of the water supply for London is one of those many-sided problems which requires to be regarded from various aspects. It has been broadly said by many writers, and if we take it as a mere expression of averages, it is true that the Thames furnishes water enough and to spare not only for the London of to-day, but for any sort or size of London which one can imagine arising for many years to come. But when we put averages on one side and compare the flow of the Thames on individual days, with the wants of London on those days, we see how very near the edge we are going in trusting to the river, and how absolutely necessary it is, unless we go to other and more distant sources, that the extensive system of storage reservoirs which has been begun should be carried to completion without undue delay. According to the report of the Local Government Board just issued it appears that although during a month when the river is full, as it is in February, the amount withdrawn from the Thames may not amount to more than 2·6 per cent. of the whole quantity, the matter is very different during the dry season when the amount withdrawn may amount to as much as 64·8 per cent. as happened in September, 1899. During last year the average takings from the Thames in September amounted to only 38·1 per cent. of the total flow, but, even in that favourable month of a favourable year, on one day, namely, on September 24th, a good deal more than half of the total flow down the Thames was abstracted for the use of London. This ought to show how nearly we have approached to the limits of our natural water supply, and how important it is that measures should be pushed forward as quickly as may be for storing some of the excess of water which during so many days of the year runs to waste down the bed of the river. The urgency of the matter in regard to some of the companies may be judged of by the statement which we cull from the Local Government Board's Report, that "the subsidence reservoirs of the Grand Junction Company contain the equivalent of only 2·9 days' consumption." It must always be remembered that storage of water not only gives security in regard to continuity of supply, but is of the greatest importance in maintaining its purity, partly because by the possession of a mple storage the companies would be able to exc1ude the foul washings of the land which are brought down by storm water, and partly because by aid of subsidence and by the mutually destructive action of water organisms such stored water undergoes a great purifying process even before arriving at the filters.

### The Medicinal Uses of Lager Beer.

For a long time back champagne has been a highly esteemed remedy, and although one may suspect that in some instances it has been given rather as a placebo and a proof of sympathy than from any great faith in its efficacy, it still occupies the first place in the minds of many as a means of giving relief in cases of vomiting. An American physician[1] has, however, lately fallen back upon a similar but somewhat cheaper remedy, namely, lager beer. He says that the acute vomiting which is frequently encountered in female pelvic disorders, in nervous diseases, and in acute general infections is a severe symptom in arresting which lager beer is very useful. Its action is certain, intolerance of it is rare, and its after effects are soporific. Of the two varieties of lager—the pale and the dark—the latter is to be preferred, and as to quantity a bottle or two suffices. He adds that singularly good results can be obtained from small doses. The beneficial effects are most marked in women and in those who are not habitual consumers of alcohol. In the nausea, bilious vomiting, vertigo, and sleeplessness, which often occur in women suffering from influenza, a wineglassful of beer repeated in half an hour will immediately sooth the disturbed organ and give a night of refreshing sleep. Accepting fully the truth of Dr. Kolipinski's clinical observations, if one endeavours to correlate his statement about lager with other methods of treating acute vomiting, one recognises at once that in many cases much relief is often obtained by having, as the stewards on board ship tell us, "something to vomit on"—something to dilute acrid juices, and dislodge mucus from the gastric mucous membrane, and also something for the contracting stomach muscle to grip without actually wringing itself into a ball. Hence much of the efficacy of weak tea, hot water, brandy or whisky and soda, and champagne, to which we must now add lager. We have no doubt about the efficacy of the latter, but beer, without the qualifying adjective, is no new thing in the treatment of disease.

[1] New York Medical News, Oct. 5.

### The Workmen's Compensation Act.

A curious anomaly in the Workmen's Compensation Act was brought to light in a case which recently came before Judge Emden at the Tunbridge Wells County Court. A firm called Tinkers had secured the contract to enlarge the electricity works of the Corporation of Tunbridge Wells, and one of their workmen named Jones received a shock from a "live" wire while engaged in removing some scaffolding. He fell from a height of 30 feet to the ground, fracturing his thigh and sustaining other injuries. He now brought an action for £50 against the Corporation and against Tinkers, Limited, under the Compensation Act. The counsel for the Corporation contended that they were not carrying out the work themselves and therefore were not liable, because Jones was not in their employ. Tinkers, Limited, on the other hand, contended that the work the man was engaged upon was not an occupation, strictly speaking, which was specified by the Act, and further that the works where the accident occurred were not their engineering works or factory. His Honour ruled that neither the Corporation nor the contractors were liable and dismissed the summons, but said it was most unfortunate for the workman and hoped that something would be done for him. The case involves an important point of principle for all corporate bodies, contractors, and working men, and it is difficult to see any way out of the difficulty. It is obvious that corporations cannot be held liable for any extra expense due to accidents at works let out to a contractor, but it is very hard on working men if they are not entitled to compensation which they would receive otherwise. The wording of the Act is indistinct, and Judge Emden by his decision has established a case which will no doubt be frequently quoted as a precedent.

